# Insights into the Genome Sequences of an *N*-Acyl Homoserine Lactone Molecule Producing Two *Pseudomonas* spp. Isolated from the Arctic

**DOI:** 10.1128/genomeA.00767-16

**Published:** 2016-08-04

**Authors:** Akhilandeswarre Dharmaprakash, Dinesh Reghunathan, Krishnakutty C. Sivakumar, Manoj Prasannakumar, Sabu Thomas

**Affiliations:** a Cholera and Biofilm Research Lab, Rajiv Gandhi Centre for Biotechnology, Trivandrum, Kerala, India; b Genomics Core Facility, Rajiv Gandhi Centre for Biotechnology, Trivandrum, Kerala, India; c Distributed Information Sub-Centre (Bioinformatics Centre), Rajiv Gandhi Centre for Biotechnology, Trivandrum, Kerala, India

## Abstract

We report for the first time the draft genome sequence of two psychrotrophic *Pseudomonas* species, *Pseudomonas simiae* RGCB 73 and *Pseudomonas brenneri* RGCB 108, from the Arctic that produce more than one acyl homoserine lactone molecule of varied *N*-acyl length. The study confirms the presence of a LuxR-LuxI (type) mediated quorum-sensing system in both the *Pseudomonas* species and enables us to understand the role of quorum sensing in their survival in extremely cold environments.

## GENOME ANNOUNCEMENT

The polar region of the Arctic is one of the least explored areas in our planet and is predominantly colonized by psychrophilic microorganisms (1, 2). The role of quorum-sensing mechanisms in psychrophiles has not yet been investigated in detail. In Gram-negative bacteria, quorum sensing is mediated by *N*-acyl-l-homoserine lactone (AHL) signal molecules and homologs of LuxI and LuxR proteins. LuxI-type synthase proteins synthesize AHLs, which interact with LuxR-type regulator proteins to regulate the gene expression of a specific set of genes related to a particular phenotype (3). In the present study, bacteria collected from the environmental samples from Ny-Alesund, Arctic at 79°N, during the Indian Arctic Expedition in 2009 were screened for the production of AHL molecules using the AHL reporter strains *Chromobacterium violaceum* CV026, *Escherichia coli* pJBA132, and *Pseudomonas putida* F117 pKR-C12, which sense short and long acyl chain AHL molecules (4, 5). Of the bacterial isolates screened, 9.6% were found to produce diverse AHL molecules. Of these isolates, two strains, *Pseudomonas simiae* RGCB 73 and *Pseudomonas brenneri* RGCB 108, were selected for whole-genome shotgun sequencing because of their varied AHL production in different temperature conditions.

Total genomic DNA was isolated using a Wizard genomic DNA purification kit (Promega), which was used for the construction of a barcoded genomic library per the manufacturer’s protocol. Final barcoded libraries were loaded onto a 318 v2 chip provided by Ion Torrent and sequenced using the Ion Torrent PGM platform. In total, 5.59 million reads were generated, providing 1.16 Gb of data which were sorted based on their bar codes, resulting in 2.56 million reads and 2.83 million reads for strains RGCB 73 and RGCB 108, respectively. Both the strains were sequenced with mean depth coverage above 50× and showed G+C content of ~60%. For strain RGCB 73, the reads were assembled using SPAdes v 3.1 (6), which resulted in 95 contigs with a total sequence length of 6,037,480 bp and an *N*_50_ of 130,892 bp. The longest contig was 501,400 bp. Similarly, for strain RGCB 108, the reads were assembled using SPAdes v 3.1, which resulted in 68 contigs with a total sequence length of 6,282,598 bp and an *N*_50_ of 217,290 bp. The longest contig was 618,143 bp. Annotation was added for both the genomes by the NCBI Prokaryotic Genome Annotation Pipeline (PGAP), released in 2013.

The PGAP predicted 5,584 coding sequences along with 70 RNA-encoding genes for strain RGCB 73 and 5,399 coding sequences along with 77 RNA-encoding genes for strain RGCB 108. In the present study, both of the strains were found to harbor the gene LuxR family transcriptional regulator, which is associated with AHL-mediated quorum sensing in their draft genomes. Additionally, many genes involved in cold adaptation were identified in both the genomes (7, 8). Genomic information gathered here will enable us to understand the role of quorum sensing-mediated gene regulation in bacteria thriving in such extremely cold environments and its role in regulating genes involved in cold adaptation.

### Accession number(s). 

The whole-genome shotgun projects of the two strains have been deposited at DDBJ/EMBL/GenBank under the following accession numbers: LUXZ00000000 and LVWZ00000000. The versions described in this paper are LUXZ00000000.1 and LVWZ00000000.1.
